# Structural annotation of acylcarnitines detected in SRM 1950 using collision-induced dissociation and electron-induced dissociation

**DOI:** 10.1007/s00216-025-06234-y

**Published:** 2025-11-20

**Authors:** Valentina Ramundi, Michael Witting

**Affiliations:** 1 Metabolomcis and Proteomics Core, Helmholtz Zentrum München, Ingolstädter Landstraße 1, 85764 Neuherberg, Germany; 2 Chair of Analytical Food Chemistry, TUM School of Life Sciences, Technical University of Munich, Maximus-Von-Imhof-Forum 2, 85354 Freising-Weihenstephan, Germany

**Keywords:** Acylcarnitines, Electron-induced dissociation (EID), High-resolution mass spectrometry, Structural elucidation, Untargeted metabolomics

## Abstract

Acylcarnitines are important intermediates in fatty acid metabolism, shuttling acyl groups into mitochondria for β-oxidation and energy production. As biomarkers, their concentrations are utilized to diagnose metabolic and cardiovascular diseases, insulin resistance, and neurodegenerative disorders. However, in-depth structural characterization of acylcarnitines is limited by conventional collision-induced dissociation (CID), which yields fragments predominantly from the carnitine headgroup with minimal information regarding the fatty acyl chains. In this case, we employed a novel quadrupole time-of-flight (QToF) mass spectrometer, the Sciex ZenoTOF 8600, with CID and electron-induced dissociation (EID) to carry out in-depth analysis of acylcarnitines detected in National Institute of Standards and Technology (NIST) SRM 1950 reference plasma. With increased sensitivity and reduced accumulation times (95 ms) on the 8600 platform, we were able to confidently annotate 35 acylcarnitines, including isomeric species and functional groups such as hydroxylations and double bonds. EID provided comprehensive structural information, enabling the discrimination of isomers such as valeryl-, isovaleryl-, and 2-methylbutyryl-carnitine (Car 5:0), as well as the positioning of methyl branching via diagnostic fragments. Hydroxylated species, such as Car 16:0;3OH, were confirmed by the presence of diagnostic ions and matched to reference standards by retention time. Moreover, EID enabled the localization of double bonds within unsaturated species (e.g., Car 18:1, Car 18:2) via fragmentation patterns that are indicative of unsaturation positions, following established lipid fragmentation mechanisms. This work demonstrates that EID offers significant advancements for the structural elucidation of acylcarnitines, delivering enhanced sensitivity and deeper insights into isomeric and functional diversity.

**Graphical abstract:**

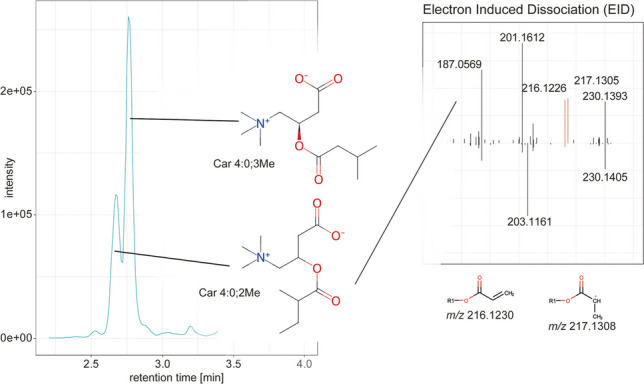

**Supplementary Information:**

The online version contains supplementary material available at 10.1007/s00216-025-06234-y.

## Introduction

Acylcarnitines are esters formed through the conjugation of fatty acids with carnitine. Their primary biological role is to facilitate the transport of acyl groups from the cytosol into the mitochondrial matrix, where these groups are transferred to coenzyme A (CoA) to form acyl-CoA, which undergoes β-oxidation, a vital metabolic pathway for energy production. This process is essential for sustaining cellular activity and energy [1]. During β-oxidation, fatty acyls are broken down into acetyl-CoA, which then can enter the Krebs cycle, ultimately leading to the generation of ATP through oxidative phosphorylation. Therefore, the transport of acyl groups by acylcarnitines is a key step in cellular energy production from fatty acids.

Since energy production is closely linked to various physiological processes, acylcarnitine levels can serve as valuable biomarkers for a wide range of metabolic disorders and diseases. Their role in transporting fatty acids for β-oxidation means that disruptions in this metabolic pathway can be reflected in the profile of acylcarnitine species, making them valuable indicators for diagnosing conditions. In rare inherited disorders, such as fatty acid oxidation disorders (FAODs), both long- and medium-chain acylcarnitines are essential for identifying defects in fatty acid metabolism. These metabolites also play a key role in diagnosing insulin resistance, as plasma concentrations of long-chain acylcarnitine reflect tissue-specific changes during fed and fasted states [2]. In addition, incomplete metabolism of long-chain acylcarnitine is a recognized marker for heart failure and a predictor of major cardiovascular events. Heart tissue is believed to be a primary contributor to the plasma long-chain acylcarnitine pool, underscoring the importance of these measurements in diagnosing cardiac conditions associated with mitochondrial dysfunction [3]. Elevated levels of circulating long-chain acylcarnitine have been consistently observed in patients with chronic heart failure [4–7]. There is also increasing interest in the role of acylcarnitines in neurodegenerative and neuropsychiatric disorders. Numerous studies have reported altered acylcarnitine profiles in conditions such as schizophrenia, Alzheimer’s disease, and Parkinson’s disease [8–11]. However, it remains uncertain whether these changes in circulating acylcarnitine levels directly correspond to those in brain tissues, or if they suggest potential crosstalk between plasma and neurological states in these disorders.

Given their crucial role in various cellular energy metabolism pathways and their increasing recognition as biomarkers in different pathological conditions, there is an ever-increasing demand for a more detailed analysis of acylcarnitines. Similar to other fatty acyl–derived metabolite/lipid species, multiple isobaric and isomeric structures exist. Therefore, in-depth structural characterization of acylcarnitines has become an important topic, as the position of functional groups, such as hydroxyl groups and double bonds, may differ based on the biological origin of the molecule.

Tandem mass spectrometry (MS/MS), whether used in flow injection analysis or coupled with liquid chromatography, is the premier tool for analyzing acylcarnitines. However, collision-induced dissociation (CID) only offers limited structural information. Using CID, only a single C–O bond breakage is observable, resulting in different fragments based on the fatty acid chain length. In positive ion mode collision-induced dissociation (CID), acylcarnitines exhibit a conserved fragmentation pattern that facilitates their identification in complex biological samples [12].

Recently, electron-induced dissociation (EID) at higher energies, also called electron-activated dissociation (EAD) or electron impact excitation of ions from organics (EIEIO), has been suggested as a suitable alternative to CID for in-depth lipid annotation [13, 14]. For example, 120 lipids from the National Institute of Standards and Technology (NIST) SRM 1950 have been structurally characterized using EID [15]. We therefore argue that EID might also be an interesting approach for the structural elucidation of acylcarnitines.

However, accumulation times for good-quality EID spectra were rather high (up to 1 s). Hence, in-depth structural characterization using EID is therefore often decoupled from initial metabolomic analysis. Therefore, in this work, we utilized a novel QToF instrument with improved sensitivity to evaluate its performance regarding the structural elucidation of acylcarnitines found in the SRM 1950 reference plasma. By combining CID and EID, we were able to annotate 36 acylcarnitines at a detailed level, including the positions of double bonds and hydroxyl groups with a reduced accumulation time of 95 ms, which enables in-depth structural characterization of acylcarnitines at LC speed during profiling analysis.

## Material and methods

### Chemicals

Acylcarnitine standards were purchased from Sigma-Aldrich Chemie GmbH (Eschenstrasse 5 D-82024 Taufkirchen) including acylcarnitines mix 1 (C0, C2) solution, product number A-141, mix 2 (C4, iC4, C5, iC5, C6, C8, C10, C12, C14, C18) solution, product number A-144, and mix 3 (C8:1,C10:1,C12:1,C14:1,C14:2,C16:1,C18:1,C18:2) solution, product number A-145. Hydroxy acylcarnitines were purchased separately from Merck, including 3-hydroxydodecanoyl-l-carnitine (52,002), 3-hydroxytetradecanoyl-l-carnitine (91,503), 3-hydroxyhexadecanoyl-l-carnitine (52,413), and 3-hydroxyoctadecanoyl-l-carnitine (49,853). Additionally, LC-MS grade water was supplied by Sigma-Aldrich Chemie GmbH, while LC-MS grade acetonitrile was sourced from CHEMSOLUTE (Dornierstrasse 4–6 71272 Renningen). Formic acid, for LC-MS, was obtained from Fisher Chemical (One Reagent Lane, Fair Lawn, NJ, 07410).

### Sample preparation

One hundred microliters of SRM 1950 plasma was mixed with 400 µL MeOH. After quick vortexing, the samples were shaken for 10 min at 1000 rpm, the samples were centrifuged for 10 min at 15,000 rpm, and the supernatant was transferred to a fresh reaction tube and evaporated to dryness in vacuo. The residue was redissolved in 50 or 100 µL of 80% H_2_O/20% ACN. The solution was transferred to an LC-MS vial for analysis.

### LC-MS analysis

Reversed-phase LC-MS analysis was performed using an Agilent Infinity II 1290 UHPLC. Acylcarnitines were separated using a Phenomenex Kinetex C18 column (100 mm × 2.1 mm, 1.7 µm particle size) and a linear gradient from eluent A (100% H_2_O + 0.1% formic acid) to eluent B (100% ACN + 0.1% formic acid). The following gradient was used: 95/5 at 0 min, 95/5 at 1.5 min, 0.1/99.9 at 10.0 min, 0.1/99.9 at 12.0 min, 95/5 at 12.1 min, and 95/5 at 15.0 min. The flow rate was set to 0.5 mL/min, and the column temperature was maintained at 40 °C. Analysis has been performed using data-dependent acquisition (DDA) in positive ionization mode on a Sciex ZenoTOF 7600 with a Turbo V ion source and Sciex ZenoTOF 8600 with an OptiFlow Pro ion source using CID and EID with the ZenoTrap on. Exact parameters for MS analysis are provided in SI Table 1. The same UHPLC system was used in combination with both MS instruments.

### Data analysis

Recently detected acylcarnitines reported to be present in SRM 1950 were used as reference [16]. Exact *m*/*z* was used to search data from the two LC-MS setups for candidate peaks in CID within the SciexOS Explorer module. For confirmation, fragmentation spectra were used. Peak areas and heights were determined using the SciexOS Analytics module. Chromatograms and reference MS^2^ spectra have been exported as text files for further processing and plotting in R. Spectra were read from the text files and internally handled as Spectra objects using the Spectra package [17]. Noise subtraction from spectra has been performed using a custom function. To validate identifications, matches were compared with our acylcarnitines and reference standards as well as EID fragmentation patterns reported by Gao et al*.* [18]. All plots were generated using the ggplot2 package in R.

## Results and discussion

### Fragmentation of acylcarnitines in CID and EAD

Fragmentation of acylcarnitines by CID has been studied in much detail, e.g., by van der Hooft et al*.* [12, 19]. The primary fragmentation event involves cleavage of the ester C–O bond between the acyl chain and the carnitine backbone, resulting in the neutral loss of trimethylamine (59.0735 Da) and generation of a dominant [M-C_3_H_9_N]^+^ ion. This is typically one of the most abundant product ions and serves as a hallmark of acylcarnitine structure. The corresponding acylium ion [RCO]^+^ is also observed and provides direct information on the fatty acid chain length and degree of unsaturation. Additional diagnostic fragments originate from the carnitine moiety, most notably at *m*/*z* 162.1125 ([M+H]^+^ of carnitines) and *m*/*z* 144.1019, corresponding to C₇H₁₄NO₂⁺ (carnitine–H₂O). The *m*/*z* 144.1019 fragment arises from cleavage at the 5th bond of the carnitine structure. Although its relative abundance is typically low (less than 20% of the base peak), it is considered relatively stable. It can persist at higher collision energies, up to 70 V or more in the case of long-chain acylcarnitines. A prominent low-mass fragment at *m*/*z* 85.0284, corresponding to the trimethylammonium propionic acid moiety, is consistently detected and serves as a reliable marker for carnitine-containing compounds. However, detailed analysis to date has focused on the identification and characterization of fragments derived from the carnitine head group. Different acyl chains are typically detected as neutral losses. Therefore, detailed structural elucidation of acyl chains is not possible, and identification is often limited to matching reference standards with commercial standards, e.g., via chromatographic retention times.

EID offers a promising solution for the detailed structural elucidation of diverse lipid classes, recently emerging as a robust alternative for lipid characterization. Building on this, we aimed to develop an advanced MS/MS method for the enhanced characterization of acylcarnitines using EID. Compared to CID, EID provides more comprehensive structural information, facilitating improved lipid analysis in both positive and negative ionization modes. Since acylcarnitines consist of fatty acids bound to a carnitine unit, EID proves to be a valuable technique for characterizing their fatty acid chains. EID generates a diverse array of chain fragments derived from the intact precursor ion, for example, with the sequential loss of CH_2_ units from saturated fatty acid backbones.

Indeed, EID has been recently used for the characterization of three isomers of Car 5:0:valeryl carnitine (Car 5:0), isovaleryl carnitine (Car 4:0;3Me), and 2-methylbutyryl carnitine (Car 4:0;2Me) [18]. They demonstrated that EID on the ZenoTOF 7600 system effectively localizes hydroxyl and methyl branching positions in acylcarnitines. These results by Gao et al*.* indicate that the structural elucidation of acylcarnitines by EID is possible and opens up new avenues for their analysis. However, the rather long accumulation time of 1 s for collecting MS2 spectra hinders broad application, apart from structure elucidation.

Efficient detection of these acylcarnitines is crucial for diagnosing disruptions in branched-chain organic acid metabolism. Diagnosis, for example, is performed with GC-MS analysis of organic acids or MS/MS of acylcarnitines, while the latter is typically performed as direct infusion. While chromatographic separation of the isomers is possible, no differentiation is possible with current CID workflows. We therefore investigated the possibility of EID on a novel QToF instrument.

### Analysis of SRM 1950 plasma using the ZenoTOF 8600

We have analyzed acylcarnitines from SRM 1950 using the novel Sciex ZenoTOF 8600, which represents the successor model of the ZenoTOF 7600 used by Gao et al*.* [18]. Technological improvements that lead to sensitivity improvements include the use of the Optiflow Pro ion source, improved DJet and QJet ion guides, which are similar to the technology introduced on the 7500 QqQ instruments, refined accelerator and mirror components for improved transmission in the TOF region, as well as a novel four-channel optical-based detector and ADC signal capture technology. We hypothesized that the improved sensitivity of this redesigned instrument also translates to improved detection and identification of acylcarnitines.

**Fig. 1:**
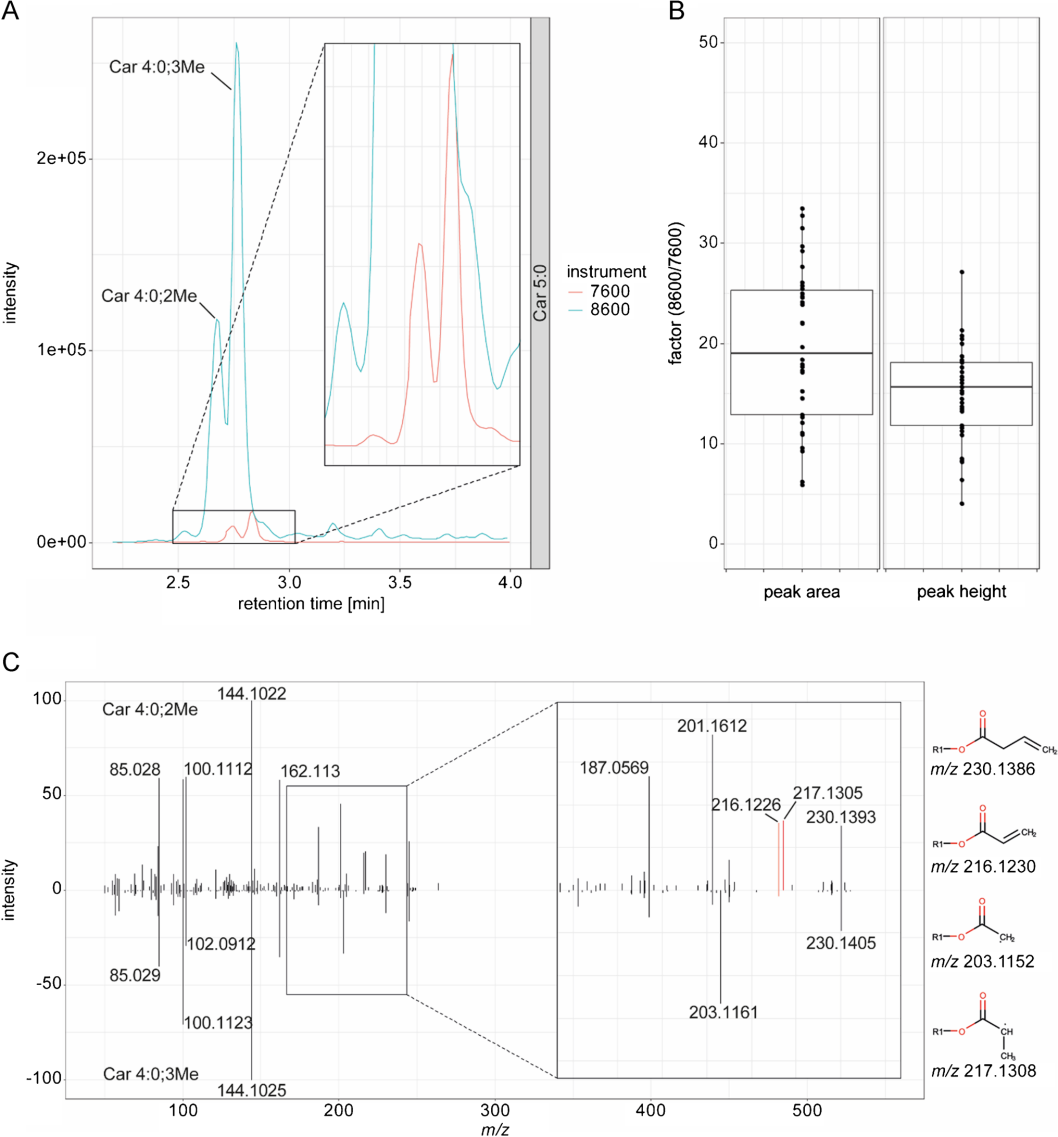
**A** Extracted ion chromatogram of Car 5:0 (*m/z* 246.17) measured on the ZenoTOF 7600 and 8600. Based on fragmentation and reference standards, the two peaks could be identified as Car 4:0;2Me and Car 4:0;3Me. **B** Boxplot of the factor of increase for peak areas or peak heights of the 8600 over the 7600. **C** Mirror plot of Car 4:0;2Me against Car 4:0;3Me. Differentiation of both is possible by the ions at *m*/*z* 216.1230 and 217.1308

First, we were interested in comparing the sensitivity of the new system and performed a comparison against the older model using the same chromatographic setup. The same LC with the same eluents was used with both the 7600 and 8600 models of the ZenoTOF. To challenge both instruments, we used the reference plasma SRM 1950 and performed measurements with both CID and EAD. All chromatographic parameters were kept constant and the same UHPLC instrument was used for both MS systems. Injection volumes were identical between the systems (5 µL). We used a recent report by Mandal et al*.* as a basis, as they have quantified 39 carnitines in this reference plasma using targeted analysis [16]. Their study was based on flow injection analysis, and therefore, no differentiation of isomeric structures was possible. On the 7600, we detected 33 different features identified as acylcarnitines, with 29 unique compositions similar to those reported by Mandal et al*.* On the 8600, we detected 35 features with 29 unique compositions. While the number of features from the HMDB list is not significantly larger on the 8600 compared to the 7600, the overall sensitivity was much higher. For example, the two peaks identified as two isomers of Car 5:0 showed a 17- and 18-fold higher peak area (Fig. 1A). Generally, the different acylcarnitines could be detected with 5-fold or more, higher peak areas and peak intensities, with a median improvement of approximately 20-fold (Fig. 1B). About 2 to 3-fold of this improvement can be attributed to the different ion sources used (OptiFlow Pro on ZenoTOF 8600 vs. Turbo V on ZenoTOF 7600). Additional improvements can be attributed to advancements in ion optics, including both novel designs and enhancements, as well as the integration of a new detector.

Next, we investigated whether the improved sensitivity translates into higher-quality EID spectra for the structural annotation of acylcarnitines. For all detected carnitine species, the ZenoTOF 8600 consistently generated higher-quality spectra compared to the ZenoTOF 7600, with improved intensity of diagnostic fragment ions essential for accurate structural elucidation. We have used a kinetic energy of 16 eV, and in contrast to Gao et al*.*, we only required 95 ms of accumulation time. We therefore used the CID and EID data obtained from the ZenoTOF 8600 for further analysis.

### Structural characterization of acylcarnitines

In the next step, we aimed to characterize the detected acylcarnitines structurally. First, for the structural elucidation of acylcarnitine, we investigated common fragments that are observed under EID conditions. Gao et al*.* described *m/z* 162.1132 and *m/z* 144.1028 corresponding to the carnitine moiety and a subsequent water loss. We also observed these fragments for different acylcarnitines, allowing them to identify acylcarnitines on a bulk level, similar to CID.

Unfortunately, we have detected contamination of MS^2^ spectra with background peaks in all EID spectra, regardless of whether they  are acylcarnitines or other metabolites. Investigating this further, we found that this contamination originates from the EID region and is likely due to one or more contaminants from the used water. However, we were able to identify all contaminant peaks and performed spectral cleaning by removing these interfering *m*/*z* values (one example is shown in SI Fig. 1).

**Fig. 2:**
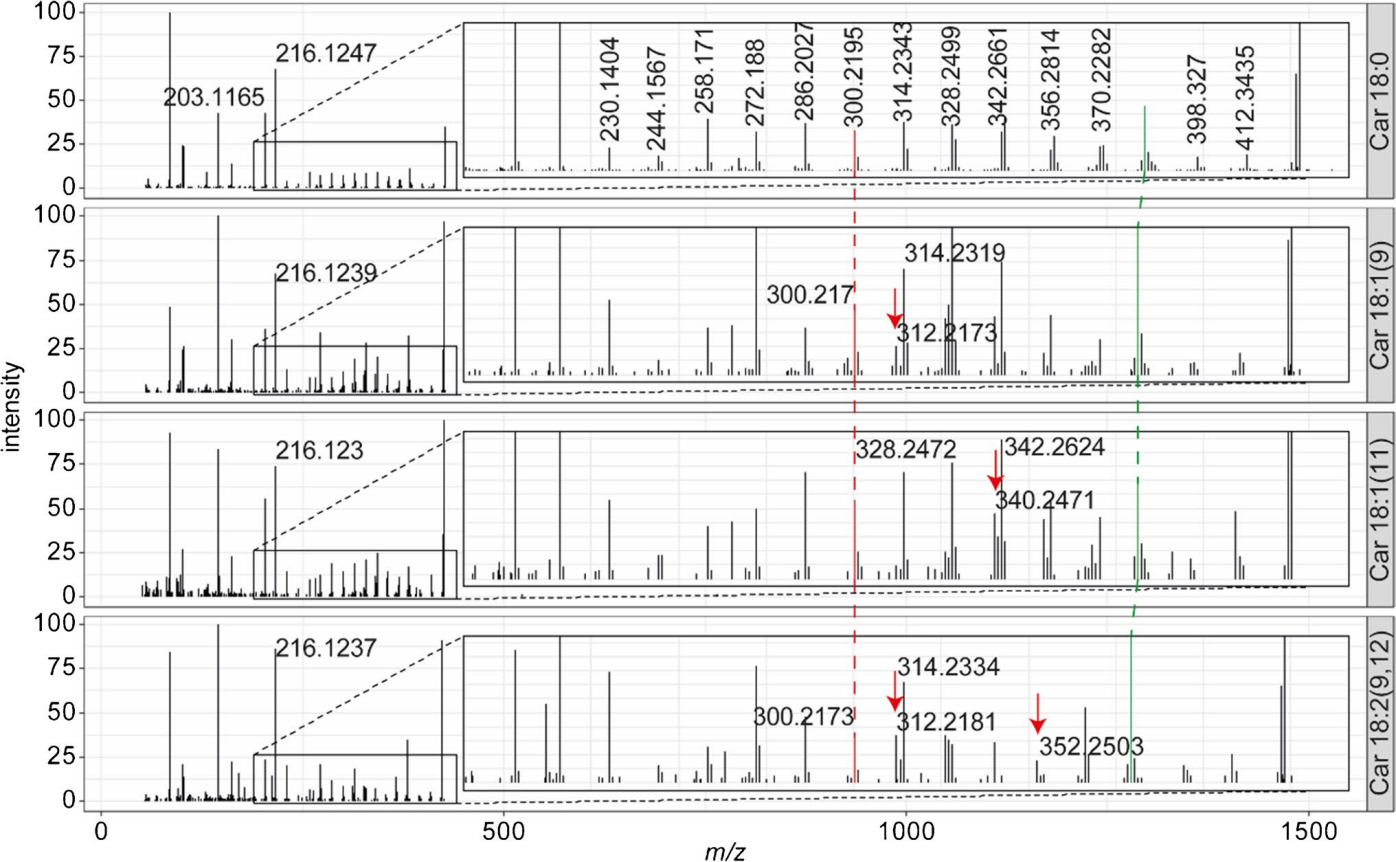
Fragmentation spectra of different acylcarnitine species with 18 carbons in the acyl chain in SRM 1950. Localization of double bonds is performed by counting from the alpha end of the acyl chain. Until *m*/*z* 300.217 (red line) all C18 species fragment the same way. For species containing a double bond at position 9 a shift of 12 Da instead of 14 is observed. The positions of double bonds at carbons 11 and 12 are identified the same way. The green line indicates the position of the CO_2_ loss, overlapping with the fragmentation pattern of the acyl chain

We first focused on the different isomers of Car 5:0, also described by Gao et al*.* [18]. Based on the two different chromatographic peaks, we could identify them as Car 4:0;3Me and Car 4:0;2Me (Fig. 1A). Both features produced a fragment at *m*/*z* 230.1386, which corresponds to the loss of the terminal CH_3_ group. Differentiation of the two isomers is possible by the fragments at *m*/*z* 203.1152, 216.1230, and 217.1308 (Fig. 1C). The structures of the fragments are shown in Fig. 2A. The fragment at *m*/*z* 217 is produced explicitly for Car 4:0;2Me. Notably, a radical ion at *m*/*z* 217.1308 appeared prominently in the Car 4:0;2Me isomer, likely due to the formation of a more stable secondary carbon radical. We have also observed this fragment in other putative 2-methylbranched chains, indicating that the combination of *m*/*z* 216.1230 and *m*/*z* 217.1308 is indicative of 2-methyl branches. In addition to Gao et al*.*, we identified the fragment at *m*/*z* 201.1723 as the decarboxylated fragment. This fragment was described by Gao et al*.* for Car 16:0;O, but we have generally observed it in different species. They described this fragment as a loss of ∙CHO_2_, but we believe this is a two-step process. First, similar to lipids, the [M+H]^+^ adduct loses an H atom, forming an [M∙]^+^ ion, and second, the loss of CO_2_. The identity of Car 4:0;3Me could be further confirmed by a chemical reference standard and matching chromatographic retention times (SI Fig. 2).

In the second step, we aimed to verify the fragmentation pattern of hydroxylated acylcarnitines, for example, Car 16:0;O, which has also been investigated by Gao et al. Besides the known fragments, an additional fragment of *m*/*z* 232.1179 is observed for 3-hydroxyl acylcarnitines. Likewise, the intensity of the fragment at *m*/*z* 216.1230 is diminished. We confirmed this fragmentation by examining fragments derived from a feature annotated as Car 16:0;O as well as a reference standard for Car 16:0;3OH, including matching chromatographic retention times.

After confirming the fragmentation pattern established by Gao et al*.*, we further investigated the acylcarnitine species described by Mandal et al*.* First, we checked the two features annotated as Car 4:0. Though their fragment intensities were generally of low intensity due to the low intensity of the peak, in the case of the first peak, we observed *m*/*z* 216.1230 and *m*/*z* 217.1308 indicating a 2-methyl branch identifying this as isobutyryl carnitine (Car 3:0;2Me) and the second feature as butyryl carnitine (Car 4:0). Additionally, retention times of reference standards fit and confirmed the fragmentation pattern.

In lipids, EID allowed the location of double bonds [13–15, 20, 21]. Based on the detection of unsaturated acylcarnitines, we investigated whether this is also possible for this class of molecules. We compared the fragmentation patterns of the features identified as Car 18:0, Car 18:1, and Car 18:2. All features could be confirmed as acylcarnitines in EID by the presence of *m*/*z* 144.1019 and *m*/*z* 162.1132. Similar to lipids and the observation of the two different Car 5:0 isomers, first the [M+H]^+^ and [M∙]^+^ ions are formed, from which further fragments are derived. For all three, a prominent peak indicating the loss of CO_2_ can be observed (*m*/*z* 383.3772 for Car 18:0, *m*/*z* 381.3594 for Car 18:1, and *m*/*z* 379.3449 for Car 18:2). The use of a kinetic energy of 16 eV led to some hydrogen rearrangements, which only occur if a double bond is present. Therefore, double bond positions are best identified by counting from the lowest observed fragment for the acyl chain, typically *m*/*z* 216.1230. A similar pattern to that of glycerophospholipids is observed, with a delta of 14 Da (CH_2_) for a single bond and 13 Da for a double bond, or sometimes 26 Da if the peaks are of low intensity. Figure 2 compares the fragmentation pattern of the observed species with 18 carbons. Table 1 summarizes the results of the identification using CID and EID. Some peaks were of low intensity and did not generate enough fragments for detailed identification.

**Table 1:** Acylcarnitines identified based on the list from Mandal et al. [16]. Retention times with a * indicate confirmation by reference standards. The different columns indicate the level which the acylcarnitines could be identified using CID and EID

Formula	*m/z*	RT (min)	Shorthand (CID)	Shorthand (EID)	Comment
C_7_H_15_NO_3_	162.1125	0.43*	Car 0:0	Car 0:0	
C_9_H_17_NO_4_	204.123	0.46*	Car 2:0	Car 2:0	
C_10_H_19_NO_4_	218.1387	0.63	Car 3:0	Car 3:0	
C_12_H_23_NO_5_	262.1649	0.70	Car 5:0;O		Very low
C_11_H_21_NO_4_	232.1543	1.23	Car 4:0	Car 3:0;2Me	
C_11_H_21_NO_4_	232.1543	1.35	Car 4:0	Car 4:0	
C_12_H_23_NO_4_	246.17	2.71	Car 5:0	Car 4:0;2Me	
C_12_H_23_NO_4_	246.17	2.80*	Car 5:0	Car 4:0;3Me	
C_21_H_37_NO_5_	384.2744	2.85	Car 14:2;O	Car 14:2;3OH	
C_21_H_39_NO_5_	386.2901	2.98	Car 14:1;O	Car 14:1;3OH	
C_13_H_23_NO_4_	258.17	3.18	Car 6:1		Very low
C_17_H_29_NO_4_	312.2169	3.68	Car 10:2		Very low
C_13_H_25_NO_4_	260.1856	3.69*	Car 6:0	Car 6:0	
C_17_H_29_NO_4_	312.2169	4.55	Car 10:2		Very low
C_15_H_29_NO_4_	288.217	4.79*	Car 8:0	Car 8:0	
C_16_H_31_NO_4_	302.2326	4.98	Car 9:0	Car 8:0;2Me	
C_17_H_29_NO_4_	312.2169	4.98	Car 10:2		Very low
C_17_H_31_NO_4_	314.2326	5.30	Car 10:1	Car 10:1(5)	
C_17_H_31_NO_4_	314.2326	5.43	Car 10:1	Car 10:1(4)	
C_17_H_33_NO_4_	316.2482	5.63*	Car 10:0	Car 10:0	
C_19_H_35_NO_4_	342.2639	5.99	Car 12:1	Car 12:1(5)	
C_23_H_41_NO_5_	412.3057	6.26	Car 16:2;O	Car 16:2;3OH	
C_21_H_37_NO_4_	368.2795	6.30	Car 14:2	Car 14:2(5,8)	
C_19_H_37_NO_4_	344.2795	6.32*	Car 12:0	Car 12:0	
C_21_H_39_NO_4_	370.2952	6.65	Car 14:1	Car 14:1(7)	
C_23_H_41_NO_4_	396.3108	6.85	Car 16:2		Very low
C_23_H_45_NO_5_	416.3371	6.97	Car 16:0;O	Car 16:0;3OH	
C_21_H_41_NO_4_	372.3108	7.01*	Car 14:0	Car 14:0	
C_25_H_47_NO_5_	442.3527	7.17	Car 18:1;O	Car 18:1;3OH	Very low
C_23_H_43_NO_4_	398.3265	7.21	Car 16:1	Car 16:1(9)	
C_25_H_45_NO_4_	424.3421	7.40	Car 18:2	Car 18:2(9,12)	
C_23_H_45_NO_4_	400.3421	7.62	Car 16:0	Car 16:0	
C_25_H_47_NO_4_	426.3578	7.78	Car 18:1	Car 18:1(9)	
C_25_H_47_NO_4_	426.3578	7.86	Car 18:1	Car 18:1(11)	
C_25_H_49_NO_4_	428.3734	8.22	Car 18:0	Car 18:0	

## Conclusion

Our results show that the ZenoTOF 8600 consistently outperformed the 7600 in both CID and EID modes. In particular, the 8600 provided the highest sensitivity, with peak area increases of up to 5-fold and more for several acylcarnitines compared to the same fragmentation mode on the 7600. The ZenoTOF 8600 also delivered richer, more informative fragmentation, especially when using EID, likely due to improved ion transmission. Overall, the ZenoTOF 8600 demonstrated clear advantages in both sensitivity and fragmentation quality, making it the superior platform for targeted analysis of acylcarnitines.

Using EID, we were able to annotate several known acylcarnitines at more detailed structural levels, e.g., isomers of Car 4:0 and Car 5:0, as well as locate double bonds and the position of hydroxyl groups. Especially, the location of double bonds will be important in differentiating unsaturated acylcarnitine species. While this is completely or partially possible by chromatography, for example, separating Car 18:1(9Z) from Car 18:1(2E), different isomers, such as Car 18:1(9Z) and Car 18:1(11Z), elute very close to each other. By speeding up chromatographic separation, these two might coelute. Using EID adds another level of confidence for differentiation.

The increased sensitivity of the ZenoTOF 8600 enabled us to use a 95-ms accumulation time for the MS2 scans, making identification at LC timescales possible without the need for arbitrarily long accumulation times. This will be a considerable advantage for identifying potentially new species of acylcarnitines in future applications and diagnostic purposes.

## Supplementary Information

Below is the link to the electronic supplementary material.

Supplementary Material 1 (DOCX 242 KB)

Supplementary Material 2 (MGF 168 KB)

Supplementary Material 3 (MGF 168 KB)

## Data Availability

Raw and cleaned EID spectra of acylcarnitines in.mgf format are available in the SI of this article.
