# Supplementary material for: Structural annotation of acylcarnitines detected in SRM 1950 using collision-induced dissociation and electron-induced dissociation
Source: Anal Bioanal Chem. 2025 Nov 20;418(3):805–12. doi: 10.1007/s00216-025-06234-y (PMC12891251; doi:10.1007/s00216-025-06234-y)
Supplement: Supplementary file 1 — Supplementary Material 1 (DOCX 242 KB) [file 216_2025_6234_MOESM1_ESM.docx]

Supplemental Information:

Structural annotation of acylcarnitines detected in SRM 1950 using Collision Induced Dissociation and Electron Induced Dissociation

Valentina Ramundi^1^, Michael Witting^1,2,^*

^1^ Metabolomcis and Proteomics Core, Helmholtz Zentrum München, Ingolstädter Landstraße 1, 85764 Neuherberg, Germany

^2^ Chair of Analytical Food Chemistry, TUM School of Life Sciences, Technical University of Munich, Maximus-von-Imhof-Forum 2, 85354 Freising-Weihenstephan, Germany

* Corresponding author: Dr. Michael Witting, [michael.witting@helmholtz-munich.de](mailto:michael.witting@helmholtz-munich.de)

**SI Table** 1**:** MS settings for analysis of acylcarnitines at the ZenoTOF 7600 or 8600.

|  | **CID 7600** | **CID 8600** | **EAD Zeno 7600** | **EAD Zeno 8600** |
| --- | --- | --- | --- | --- |
| **Ion Source** | TurboV | OptiFlow Pro Analytical> 200uL/Cal | TurboV | OptiFlow Pro Analytical> 200uL/Cal |
| Curtain gas | 40 | 40 | 40 | 40 |
| CAD gas | 7 | 7 | 7 | 7 |
| Ion source gas 1(psi) | 45 | 45 | 45 | 45 |
| Ion source gas 2(psi) | 45 | 45 | 45 | 45 |
| Temperature (°C) | 500 | 400 | 500 | 400 |
| **IDA Survey experiment** |  | | | |
| Polarity | positive | positive | positive | positive |
| Spray voltage (V) | 5500 | 5500 | 5500 | 5500 |
| Mass range (Da) | 70-1500 | 70-1501 | 70-1500 | 70-1500 |
| Accumulation time (s) | 0.1 | 0.1 | 0.1 | 0.1 |
| Declustering potential (V) | 80 | 0 | 80 | 0 |
| Collision energy (V) | 10 | 10 | 10 | 10 |
| Collision energy spread (V) | 0 | 0 | 0 | 0 |
| Time bins to sum | 6 | 6 | 6 | 6 |
| Maximun candidates ion | 10 | 10 | 10 | 3 |
| Intensity threshold exceeds (counts/s) | 10 | 10 | 10 | 10 |
| **IDA Dependent** |  | | | |
| Spray voltage (V) | 5500 | 5500 | 5500 | 5500 |
| Fragmentation mode | CID | CID | EAD | EAD |
| Mass range (Da) | 50-1500 | 50-1500 | 50-1500 | 50-1500 |
| Accumulation time (s) | 0.025 | 0.025 | 0.095 | 0.095 |
| Declustering potential (V) | 80 | 0 | 80 | 0 |
| Collision energy (V) | 35 | 35 | 12 | 12 |
| Electron beam current (nA) | - | - | 7000 | 7000 |
| Electron KE (eV) | - | - | 16 | 16 |
| ETC | - | - | 100 | 100 |
| Reaction time (ms) | - | - | 30 | 30 |
| Zeno threshold (cps) | 80000 | 1000000 | 80000 | 1000000 |
| Time bins to sum | 6 | 6 | 6 | 6 |


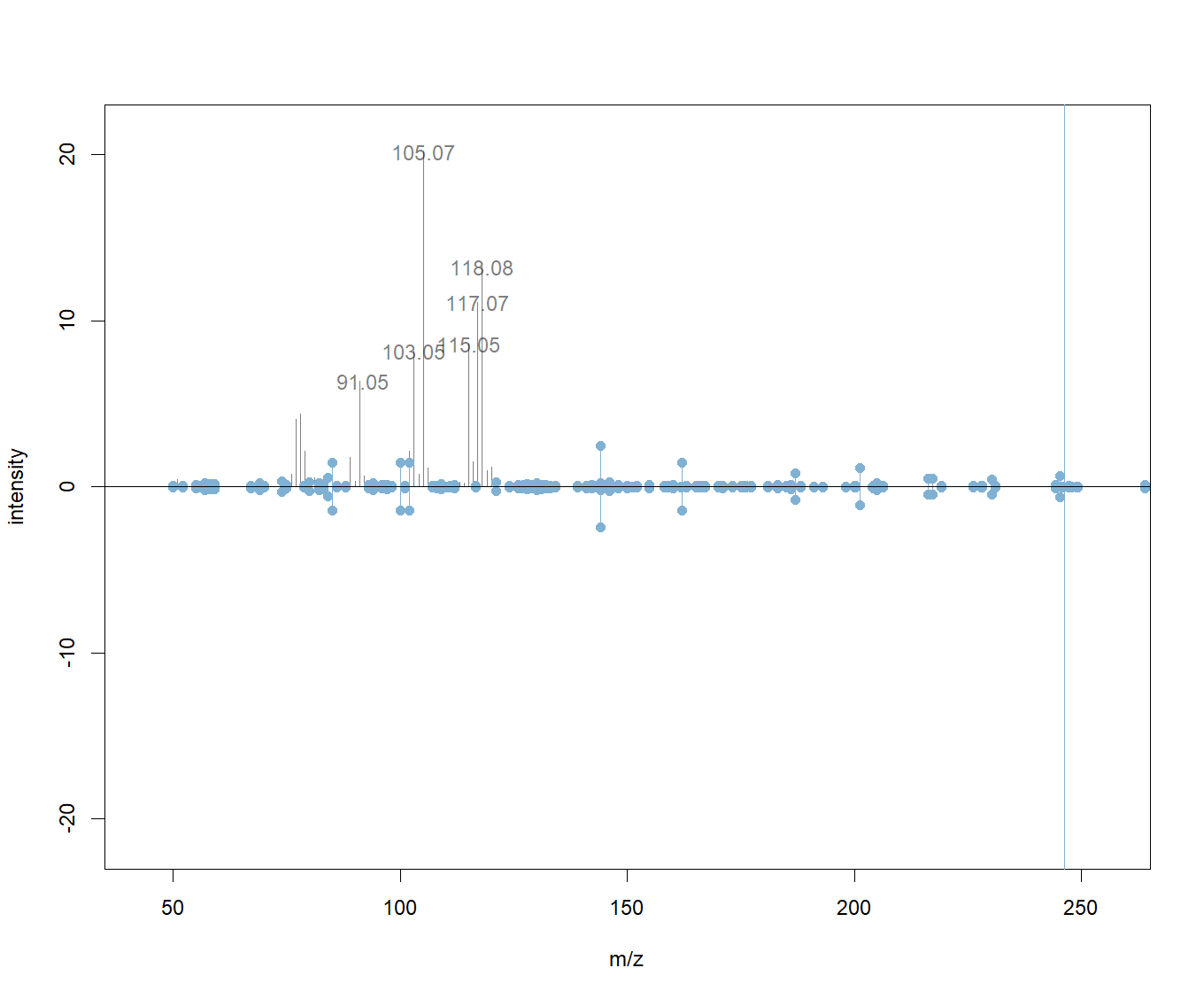


**SI Figure** 1: Example mirror plot of an uncleaned and cleaned spectrum. Contamination is observed in the lower mass region and is not interfering with the localization of double bonds. Peaks marked in blue are the remaining after spectra cleaning.


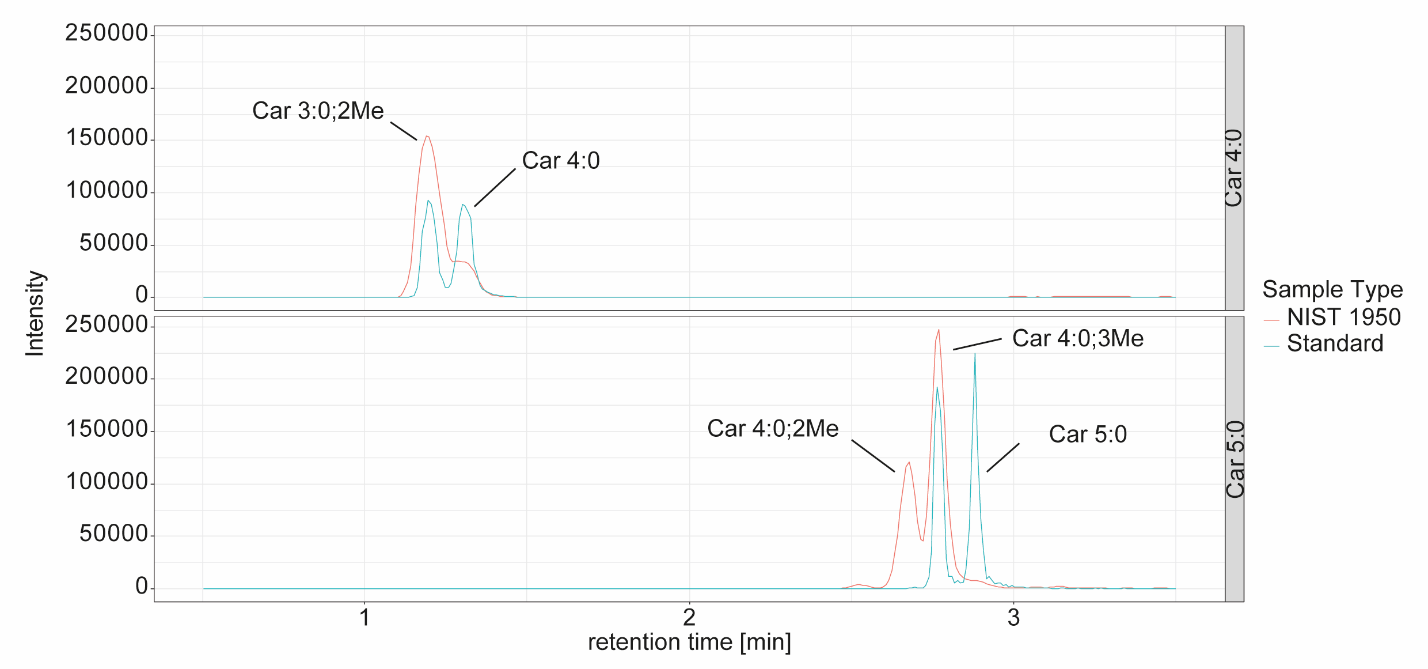


**SI Figure** 2: Extracted ion chromatograms Car 4:0 and Car 5:0 from plasma (red) and reference standards (blue). In both cases, retention times of reference standards confirm the identity suggested by EID fragmentation.
